# Late Occurrence of PML in a Patient Treated for Lymphoma with Immunomodulatory Chemotherapies, Bendamustine, Rituximab, and Ibritumomab Tiuxetan

**DOI:** 10.1155/2015/892047

**Published:** 2015-01-29

**Authors:** Michael A. Lane, Vijay Renga, Andrew R. Pachner, Jeffrey A. Cohen

**Affiliations:** Department of Neurology, Dartmouth Hitchcock Medical Center, One Medical Center Drive, Lebanon, NH 03766, USA

## Abstract

PML caused by John Cunningham (JC) virus is a rare but an increasingly recognized entity. With the advent of newer immunomodulatory therapies with monoclonal antibodies, there is an increasing incidence of PML. Initially concern was restricted to patients treated for multiple sclerosis with natalizumab but more case reports are being reported during treatment for other conditions like Crohn's disease and lymphoma with agents such as rituximab. We report the case of a 66-year-old woman who developed PML a year after completion of therapy with rituximab, ibritumomab, and bendamustine.

## 1. Introduction

A 66-year-old right handed female with history of follicular lymphoma in remission presented to the Neurology service in April 2014 with worsening left leg weakness over the previous 4 weeks associated with a foot drop.

She was diagnosed with stage IV follicular non-Hodgkin's lymphoma discovered after a workup for chest pain in August 2012, imaging revealing pulmonary nodules with biopsy revealing follicular lymphoma. She had omentectomy due to omental involvement and completed 4 cycles of bendamustine and rituximab with initial treatment followed by ibritumomab tiuxetan as part of clinical trial which she completed in April 2013. This was 12 months before development of left sided weakness. Her past medical history was significant for rheumatoid arthritis on hydroxychloroquine, coronary artery disease, hypertension, anterior cervical discectomy, and fusion.

She had a total hip arthroplasty performed 4 months prior to presentation and recovered well after surgery ambulating with a walker. Four weeks prior to presentation she developed progressively worsening left hemiparesis, left foot drop, and increasing falls. Over the same time frame she developed increased urinary frequency, incomplete voiding, urinary incontinence as well as constipation, and difficulty swallowing without overt aspiration. She denied numbness, tingling, difficulty chewing, and double vision or blurry vision. She had no fever, weight loss, or any other constitutional symptoms. There was no lymphadenopathy and no additional significant findings on the general physical exam. Neurological exam revealed an alert female appearing stated age, oriented to person, place, time, and situation with fluent and appropriate speech. She had increased tone in the left upper and lower extremity. She had 4 out of 5 strength in the left arm and 3+ out of 5 strength in left leg and 3 out of 5 strength in ankle dorsiflexion/plantarflexion. Deep tendon reflexes were brisk bilaterally, more pronounced on the left side. There was a left plantar extensor and right flexor response.

**Figure 1:**
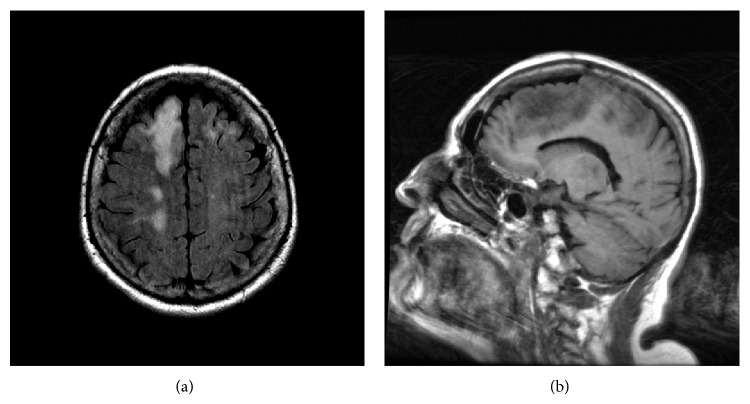
(a) T2 FLAIR: confluent hyperintense lesion in R hemisphere over R frontal region, R motor cortex. Involvement of U fibers. Minimal perilesional edema. Few lesions are seen in left frontal region as well. (b) Sagittal T1 images show hypointense lesion in the left frontal regions corresponding to the axial images.

Routine labs were within normal limits. MRI lumbar spine and EMG were normal. MRI brain showed increased flair signal intensity in the right frontal region, pons, and posterior right hemisphere without enhancement. MRIs of cervical and thoracic spine were unremarkable with postsurgical changes due to prior cervical spinal surgery (Figure 1). CSF analysis revealed a mildly elevated protein (56), normal glucose, and minimally elevated lymphocyte-predominant nucleated cells (4) with no erythrocytes. Other CSF studies include qualitative JC virus PCR, oligoclonal banding, and basic myelin protein which were negative. She was discharged with visiting rehabilitation services. The patient had progressive weakness over the course of 3 weeks and was readmitted due to recurrent falls. On neurological exam she had a new left homonymous hemianopia and a decline in strength, with inability to dorsiflex and plantarflex against gravity on the left side. A repeat MRI revealed progression of the areas of signal alteration and enhancement within subcortical white matter bilaterally increased in extent compared to the previous study. A PET CT of the head showed decreased metabolism in the regions of signal alteration identified on MRI. CSF analysis on this admission showed similar findings as previously with slightly elevated protein and mildly elevated cell count. Flow cytometry and JC virus PCR were repeated. In the anticipation of another negative test, neurosurgery was contacted to perform a brain biopsy as every other test was negative. Prior to the planned biopsy, the CSF JC virus PCR came back positive (>10 DNA copies/mL) (Mayo Medical Laboratories) as well as a positive serum anti-JCV antibody. It was felt that these results obviated the need for biopsy. The patient's exam progressively worsened during the course of the hospitalization with strength on the left side decreasing gradually. Her hydroxychloroquine utilized for management of rheumatoid arthritis was discontinued and she was sent to physical rehabilitation in the hopes that she might gain some strength back.

The patient returned for followup in June 2014 at which point her exam had improved mildly with increase in distal strength on her left side and improvement of visual field deficits and peripheral vision. She had improvement in functional status and was discharged from rehabilitation. MRI brain in August revealed mild progression of signal alteration with a decrease in cortical enhancement.

## 2. Discussion

PML is a neuroinfectious disease caused by endemic John Cunningham (JC) virus which belongs to the papovavirus family. PML cases saw a marked increase in the era of immunodeficiency from human immunodeficiency virus (HIV) infection and subsequently decreased with highly active antiretroviral therapy. Even though the vast majority of PML cases have been associated with HIV there are other conditions that predispose to JC virus activation. There has been resurgence in this condition with advances in immunomodulatory therapies. Treatment of multiple sclerosis with natalizumab received initial attention in regard to PML. Natalizumab has been reported to have in addition to the known inhibitory effect on T cells inhibitory effect on B cell functioning and this may be the reason for increased risk of developing PML [1]. Recently there have been case reports of PML occurrence in patients being treated for other conditions like Crohn's disease, lymphoma, systemic lupus erythematosus, and transplant recipients on immunosuppression.

Although PML usually occurs during immunosuppression, PML can present months to over a year after completion of immunomodulatory therapy, as described by Warsch et al. 2012 [2]. Our case is similar to the patient described by Warsch et al. in that our patient received the same three medications rituximab, bendamustine, and ibritumomab and symptoms started over a year after the completion of treatment. Etiology or pathophysiology of late onset of PML in these patients is not clear. There are over 70 cases of PML reported in patients on rituximab treatment [3]. There is no clear evidence regarding the incidence of PML in patients receiving bendamustine or ibritumomab. Since the 1990s, the vast majority of patients developing PML in the setting of lymphoproliferative disease have occurred in patients treated with purine analogues, particularly with rituximab.

Ibritumomab tiuxetan is an yttrium-90-conjugated monoclonal antibody to CD20. The anti-CD20 antibody engineered human chimeric found in rituximab is linked to tiuxetan, an MX-DTPA chelator. The added benefit of yttrium-90 radioisotope to anti-CD20 has shown an increase in overall response rate compared to rituximab in patients with B-cell non-Hodgkin's lymphomas. It utilizes targeted radiation in a single-dose outpatient schedule that is well tolerated with primarily hematologic adverse events [4]. Bendamustine is another chemotherapy agent FDA approved for rituximab refractory indolent non-Hodgkin's lymphoma. It has multiple components including an alkalizing agent with similar activity to cyclophosphamide, a benzimidazole ring which is similar to purine analogs and a nitrogen mustard for antimetabolite properties [5].

In patients with PML, a history of preexisting conditions like lymphoma causes diagnostic confusion regarding recurrence versus a new diagnosis of PML. Other diagnostic considerations generally include HIV encephalitis, vasculitis, toxoplasmosis, multiple sclerosis, acute disseminated encephalomyelitis (ADEM), or paraneoplastic processes. Unfortunately, there is a high false-negative rate (37%) of JCV serology [6] indicating that a negative antibody does not indicate absence of JCV infection. CSF analysis may help narrow the differential diagnosis. Protein is typically elevated even though it may be normal. Our patient had elevated protein on both occasions and elevated IgG index with absent oligoclonal bands in CSF suggestive of an active CNS process which was unlikely to be MS. CSF JC virus PCR is a very important test in these patients and almost as sensitive and specific as brain biopsy which is considered the gold standard. Reason for false negative CSF PCR as happened initially in our patient is thought to be due generally to low copy number of JCV and inadequate sensitivity of the PCR. Therefore a repeat CSF study would ideally be considered before brain biopsy if clinical suspicion remains high (Table 1). As low levels of JCV DNA are present in CSF (often <100 copies/mL) in patients, a complementary technique of CSF JCV antibody index may be performed [7]. Notably in this case, the JCV PCR was positive for >10 copies/mL; however, the initial negative value indicates how PML can result from only a small viral burden. Brain biopsy typically shows demyelination and vacuolation with intranuclear inclusions of viral particles which is characteristic of JC virus infection. Additional testing including in situ hybridization testing can be performed on biopsied tissue to confirm diagnosis of presence of JCV.

The majority of healthy individuals harbor antibodies to JC virus without any identifiable active viral replication. Immunity against JC virus is thought to be primarily mediated through T cells and infection with JC virus typically occurs with CD4 counts less than 200. The mechanism of reactivation of the virus secondary to monoclonal antibodies is thought to be due to inhibition of T cell endothelial adhesion, interfering with transmigration and immunosurveillance in the CNS. Natalizumab and efalizumab inhibit immune cell migration affecting T lymphocytes and however may affect B lymphocytes as well [1]; rituximab and ibritumomab specifically inhibit B lymphocytes. The fact that natalizumab may affect B lymphocytic immunity as well as reports of chemotherapy which inhibits B lymphocytes may indicate that mechanism of JCV infection may also be affected by B lymphocytic immunodeficiency.

This case has shown that concern for PML is not limited to patient's receiving immunomodulation such as natalizumab in the treatment of MS, nor patients with HIV. The use of chemotherapeutic agents, such as the medications mentioned herein, should warrant monitoring. This monitoring may include screening serum JCV antibody prior to initiating treatment, as is done prior to natalizumab initiation. Additionally, there are studies that mention that measuring anti-JCV antibody levels in serum as an index may further differentiate PML risk in MS patients that are anti-JCV positive [8]. Anti-JCV antibody is currently only used to measure risk of PML in patients with MS and is not validated in other conditions; however further investigation may result in a broader indication. Anti-JCV serology would not necessarily preclude the use of these chemotherapies and however would indicate which patients are at increased risk for developing PML thus increasing vigilance when symptoms develop.

## Figures and Tables

**Table 1:** Laboratory and diagnostic workup.

Test	Result
CSF study 1 (4/18/2014)	Cells 3, RBC 0, glucose 75, protein 56, cytology negative, JC virus PCR negative
CSF study 2 (5/9/2014)	Cells 6, RBC 3, glucose 70, protein 57, cytology negative, flow cytometry negative, **JC virus PCR positive**
HIV 1 & 2	Negative
CD4 count	144
